# TGDBN: Topology-guided dual-backbone network for semi-supervised blastocyst segmentation in assisted reproductive technology

**DOI:** 10.1371/journal.pone.0355053

**Published:** 2026-08-06

**Authors:** Yiming Li, Hua Wang, Jingfei Hu, Jicong Zhang

**Affiliations:** 1 School of Biological Science and Medical Engineering, Beihang University, Beijing, China; 2 Hefei Innovation Research Institute, Beihang University, Hefei, China; 3 School of Medical Informatics Engineering, Anhui University of Traditional Chinese Medicine, Hefei, China; 4 State Key Laboratory of Cognitive Intelligence, Hefei, China; University of Salerno: Universita degli Studi di Salerno, ITALY

## Abstract

In the field of assisted reproductive technology (ART), accurate segmentation of blastocyst tissues is crucial for evaluating embryo implantation potential. However, existing semi-supervised learning (SSL) methods often suffer from issues such as structural ambiguity, scale confusion, and insufficient utilization of unlabeled data, while the complex nested structure and inherent transparency of blastocyst images further exacerbate these challenges. To address these challenges, this study proposes a Topology-Guided Dual-Backbone Network (TGDBN), a novel semi-supervised segmentation framework featuring a *single-model dual-head architecture*: one head for pixel-level segmentation and the other for topology-guided structural optimization. Unlike traditional SSL methods that rely solely on feature consistency, TGDBN incorporates two core innovations within its topology-guided head: 1) Decoder Intra-Class Enhancement (DICE) module: Targeting the last three layers of the decoder (where fine-grained structural information is concentrated), this module adaptively strengthens class-specific “hard features” (*e.g.*, low-contrast boundaries between TE and blastocoel) through category-channel mapping and Gaussian weighting; 2) Multi-Scale Feature Aggregation (MSFA) module: This module upsamples DICE-enhanced multi-scale features to the original image resolution, concatenates them to capture hierarchical structural cues, and feeds the aggregated features into a regression sub-layer to generate explicit topology-guided structural guidance. Experimental validation on a public blastocyst dataset demonstrates that TGDBN outperforms 11 state-of-the-art SSL methods. Visualization results confirm that the network effectively mitigates boundary ambiguity and improves intra-class consistency. Further tests on the left atrial (LA) dataset shows that TGDBN exhibits robust generalization capability. These results verify that TGDBN can effectively leverage unlabeled data to enhance blastocyst segmentation accuracy and structural continuity.

## Introduction

In the field of assisted reproductive technology (ART), precise segmentation of blastocyst tissues, including inner cell mass (ICM), trophectoderm (TE), blastocoel and zona pellucida (ZP) is a critical step in evaluating embryo implantation potential, as it directly supports embryologists in assessing embryonic developmental quality [1]. However, existing semi-supervised learning (SSL) methods for blastocyst segmentation face three critical limitations: 1) structural ambiguity caused by the inherent transparency of embryonic tissues and Hoffman modulation contrast (HMC) optics (which obscure boundaries between adjacent tissues like TE and blastocoel) [2]; 2) scale confusion arising from significant depth-of-field variations in blastocyst images (leading to mismatched feature scales between superficial ZP and deep ICM) [3]; 3) insufficient exploitation of unlabeled data due to over-reliance on feature consistency constraints (failing to mine structural correlations in unannotated samples). These issues are further compounded by the complex nested structure of blastocysts, leading to suboptimal segmentation accuracy and poor boundary refinement.

Although existing studies have endeavored to enhance segmentation structural continuity via topology-related tasks (*e.g.*, Topology-constrained methods [4]), directly impose topological constraints on segmentation results through graph-based regularization, yet struggle with inadequate adaptation to complex nested structures of blastocysts. Input-embedded topology methods incorporate pre-extracted topological features (*e.g.*, Euler characteristic, Betti number) as additional inputs to the segmentation network, with representative approaches such as [5]. Nevertheless, these methods exhibit weak dynamic interaction between topological features and image features, as the pre-extraction process of topological information is independent of the segmentation network training, leading to potential feature mismatch. Furthermore, a common shortcoming of these methods is the lack of a *unified single-model framework* to coordinate segmentation and topology learning, resulting in disjointed feature optimization and inefficient structural information utilization.

**Fig 1:**
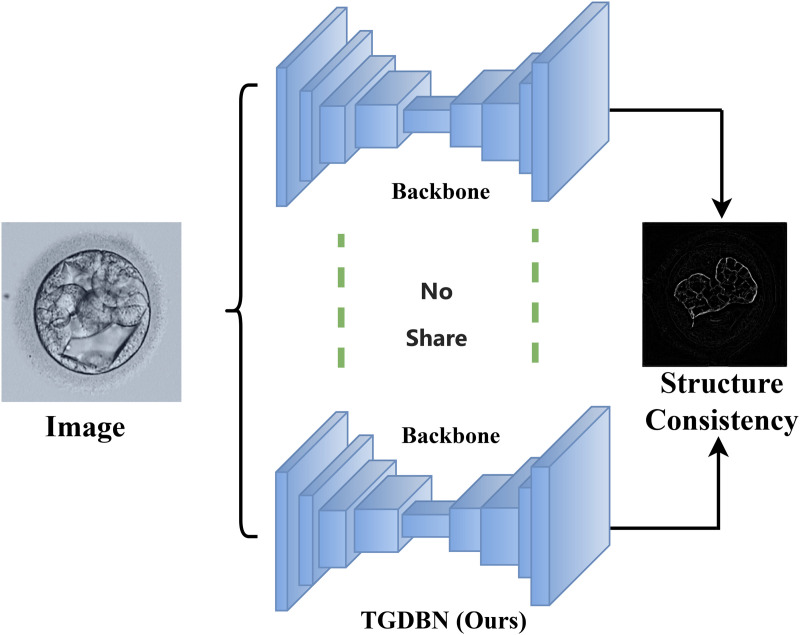
The schematic diagram of our proposed TGDBN. The network adopts a single-model dual-head architecture: the Segmentation Head outputs pixel-level tissue category predictions, while the Topology-Guided Head integrates DICE and MSFA Modules to generate structural guidance for segmentation optimization.

To address the above pain points, we propose a Topology-Guided Dual-Backbone Network (TGDBN), as shown in Fig 1. Distinct from existing dual-network or multi-branch designs, TGDBN adopts a *single-model dual-head architecture* that shares a common encoder but splits into two dedicated heads in the decoder stage: 1) *Segmentation Head*. Responsible for pixel-level blastocyst tissue segmentation, it maps encoder-extracted features to the category space via convolutional and Softmax layers, outputting tissue category probability maps; 2) *Topology-Guided Head*. Focused on structural guidance generation, it integrates two core modules to optimize segmentation from a topological perspective, and its output is used to constrain both labeled and unlabeled data learning.

The implementation logic of the Topology-Guided Head is as follows: First, to address the uneven structural features of blastocyst tissues across different depth-of-field regions, a *Decoder Intra-Class Enhancement (DICE)* Module is designed. This module targets the last three layers of the decoder (where the richest fine-grained structural information converges) and first binds feature channels to specific tissue categories via “category-channel mapping,” *i.e.*, dividing each decoder layer’s feature map into *C* category-specific channel groups to avoid cross-category interference. Then it applies a Gaussian weighting strategy to calculate adaptive weights from the Segmentation Head’s probability maps, amplifying the contribution of “hard features” (*e.g.*, blurred boundaries, low-contrast regions) while preserving reliable high-confidence features, effectively reducing intra-class feature variation. Second, to resolve scale confusion and capture multi-scale structural correlations (from local cell boundaries to global tissue topology), a *Multi-Scale Feature Aggregation (MSFA)* Module is proposed. This module first upsamples the DICE-enhanced multi-scale features (from the decoder’s last three layers) to the original image resolution via bilinear interpolation, then concatenates them along the channel dimension to integrate structural cues of different scales. The aggregated features are fed into a customized regression sub-layer (comprising two 3×3 convolutional blocks and a 1×1 convolution) to generate an explicit structural guidance map, which encodes the signed distance from each pixel to the nearest tissue topological structure to provide clear structural constraints for the segmentation task.

The main contributions of this study can be summarized as follows:

- We propose a single-model dual-head architecture for blastocyst segmentation, which unifies pixel-level segmentation and topology-guided structural optimization in one framework. This design avoids feature disjointedness in multi-network methods and enables efficient interaction between segmentation and structural information.
- Within the Topology-Guided Head, the DICE Module accurately enhances class-specific hard features through category-channel binding and Gaussian weighting, while the MSFA Module constructs a unified global-local structural representation by aggregating multi-scale features. The two modules work synergistically to improve the discriminative power of structural features and alleviate scale confusion.
- On the public blastocyst dataset, TGDBN achieves a Dice coefficient of 91.78%, outperforming 11 mainstream SSL methods. Further tests on the left atrial (LA) dataset (medical images with structural features similar to blastocysts) confirm that the network exhibits robust generalization in segmenting structurally complex medical images, providing a transferable technical solution for clinically relevant segmentation tasks.

## Related work

### Core paradigms of semi-supervised learning and adaptability to medical images

Embryo image segmentation serves as a crucial support for computational embryology and assisted reproductive technology, and its performance directly impacts the accuracy of embryo quality assessment, developmental potential prediction, and clinical decision-making. However, this field has long been constrained by the scarcity of high-quality annotated data. Medical image annotation relies on the professional knowledge of embryologists, which is not only time-consuming, labor-intensive and costly, but is also inevitably subject to subjective differences. This annotation bottleneck forms a sharp contradiction with the demand for large-scale annotated data by deep learning models [6,7]. Semi-supervised learning provides an effective solution to this contradiction by collaboratively using a small amount of annotated data and a large amount of unannotated data for model training. First, consistency regularization, which explores potential structural information in unannotated data by forcing the model to output consistent predictions for different perturbed versions of inputs; second, pseudo-labeling, which uses the model’s high-confidence prediction results on unannotated data as pseudo-labels to iteratively supplement supervision signals for model optimization; third, entropy minimization, which encourages the model to generate low-entropy (high-confidence) predictions for unannotated data to improve the clarity of decision boundaries [8]. However, these general paradigms face significant challenges when adapting to embryo images, making it difficult to effectively address domain-specific issues such as low contrast, blurred structures, and complex spatiotemporal dynamic changes.

### Research progress and existing challenges in semi-supervised segmentation of embryo images

In response to the characteristics of embryo images, researchers have made domain-specific improvements based on general semi-supervised learning frameworks, forming various targeted methods. Topology-aware semi-supervised learning integrates biological constraints and topological information of embryo development into the model, significantly improving the recognition accuracy of inner and outer membranes by ensuring that the segmentation results comply with topological rules such as cell contact relationships and layered structures. A 3D live embryo cell segmentation method proposed in 2024 further combines synthetic dataset expansion and topological constraints to effectively solve the core problem of blurred cell boundaries [9]. The geometric constraint integration strategy encodes prior knowledge of embryo morphology (such as cell size and shape regularity) in the loss function, making the segmentation results more consistent with the conservative developmental patterns of human embryos [9]. Multi-modal fusion and self-supervised pre-training integrate multi-modal data such as phase-contrast microscopy and fluorescence microscopy, explore general features through cross-modal contrastive learning, reduce the dependency on annotated data and improve model generalization ability [10]. The combination of adaptive contrast enhancement and semi-supervised frameworks realizes end-to-end joint optimization of image preprocessing and segmentation tasks, allowing for the targeted enhancement of key features without manual adjustment of parameters [8]. The introduction of multi-scale feature fusion and attention mechanisms improves the perception of blurred edges by capturing multi-level information from local details to global context, among which the hybrid architecture of Transformer and CNN performs outstandingly, capable of modeling both local intensity changes and long-range dependencies [11]. Generative data augmentation technology, especially diffusion model-based methods, can synthesize highly realistic and diverse embryo images, effectively supplementing the sample gap of rare cell types and developmental stages. A 2024 study used conditional diffusion models to generate embryo images at specific stages of development, significantly alleviating the problem of unbalanced data distribution [12–14]. Uncertainty-aware methods are specifically designed for blurred boundary regions, adjusting pseudo-labeling strategies by quantifying prediction confidence to avoid the propagation and amplification of incorrect annotations. A 2024 study showed that this method can improve the Dice coefficient of cell boundary segmentation by approximately 5–7% [7,15,16]. Dynamic reweighting strategies alleviate the problem of class imbalance in the early stages of embryo development by adjusting class weights and spatial position weights [11]. Despite the certain progress made by existing methods, most of them simply adapt general deep learning frameworks to embryo image tasks, failing to fully explore the inherent structural features of embryo images. Due to the transparent nature of embryo cells and the requirement of no staining, the images have inherent problems such as weak intensity differences between cells and the background, as well as between cells themselves, and unclear boundary definitions, leading to limited segmentation accuracy and robustness. How to deeply integrate the structural features of embryo images, enhance tissue-specific feature expression, and combine topological structure guidance to fully exploit the intrinsic value of data has become a key research direction to improve model performance.

## Method

This section provides a detailed introduction to the proposed network, with a focus on its dual-backbone dual-head architecture and the core modules within the Topology-Guided Head, as well as the corresponding loss functions.

### Overall network architecture

**Fig 2:**
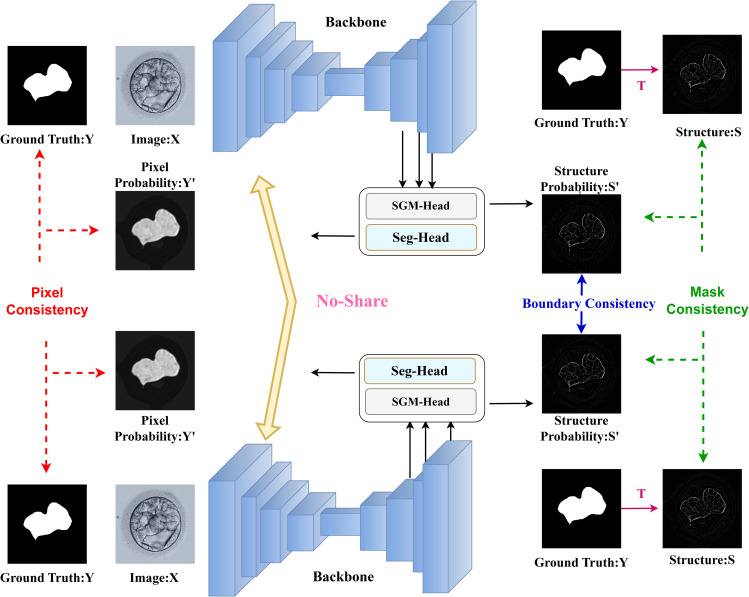
Overall dual-backbone dual-head architecture of the network. Each backbone outputs pixel probability $Y^{\prime }$ via Seg-Head and structural probability $S^{\prime }$ via SG-Head. Consistency constraints are applied between the two backbones to optimize model learning.

The network adopts a *non-shared weight dual-backbone* design, as illustrated in Fig 2. Each backbone processes the input image *X* independently and includes two functional heads: a Segmentation Head (Seg-Head) for pixel-level segmentation and a Structure Guidance Head (SG-Head, *i.e.*, TG-Head) for structural information modeling. The key role of the dual-backbone structure is to enforce consistency constraints (pixel consistency and boundary consistency) between the two branches, with a consistency mask guiding the effective application of these constraints. Ground truth data includes pixel-level segmentation label *Y* and structural label *S*.

### Backbone and Seg-Head

The segmentation head (Seg-Head) is implemented as a lightweight fully convolutional module to map high-resolution decoder features to pixel-level category predictions. Seg-Head contains convolutional blocks (3×3 convolution + BN + ReLU) and a 1×1 convolution layer. After the final convolution, a softmax activation is applied across the category dimension to generate the pixel-level probability map $Y^{\prime }\in [0,1{]}^{H\times W\times C}$, where the probability of a pixel (*i*,*j*) belonging to category *c* is given by:


$$\begin{array}{c} Y_{i,j,c}^{\prime }=\frac{\exp\left(f_{Seg}(X{)}_{i,j,c}\right)}{\sum_{k=1}^{C}\exp\left(f_{Seg}(X{)}_{i,j,k}\right)}, \end{array}$$ (1)

where $f_{Seg}(X{)}_{i,j,c}$ denotes the logit value for category *c* at pixel (*i*,*j*) output by the final 1×1 convolutional layer. The softmax normalization ensures $\sum_{c=1}^{C}Y_{i,j,c}^{\prime }=1$ for all pixels.

### SG-Head (topology-guided head)

The nested anatomical structure of blastocysts-where the ICM is enclosed by the TE, which itself surrounds the blastocoel, all within the ZP-presents a natural topological prior that should be preserved during segmentation. While existing SSL methods enforce pixel-level consistency, they often fail to capture such structural regularity, leading to anatomically implausible predictions (*e.g.*, discontinuous TE layers or misaligned tissue boundaries). To address this, we propose an explicit topological guidance mechanism that translates biological nesting into a learnable geometric representation, enabling the model to maintain anatomical consistency even when labeled data are scarce.

**Fig 3:**
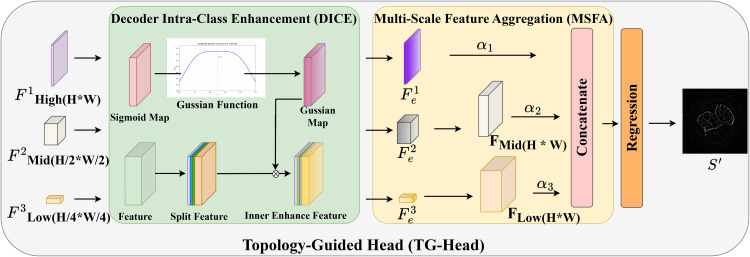
Detailed structure of SG-Head. The DICE module enhances multi-scale decoder features using Gaussian functions. The MSFA module aggregates enhanced features and generates $S^{\prime }$ via regression.

The SG-Head (as shown in Fig 3) is dedicated to generating structural guidance information, integrating two core modules: Decoder Intra-Class Enhancement (DICE) Module and Multi-Scale Feature Aggregation (MSFA) Module. Its input is the last three layers of the decoder (High-scale: H×W, Mid-scale: H/2×W/2, Low-scale: H/4×W/4), and its output is the structural probability map $S^{\prime }\in [0,1{]}^{H\times W}$, which encodes the relevance of each pixel to tissue boundaries.

#### Decoder intra-class enhancement (DICE) module.

The DICE module enhances class-specific “hard features” (*e.g.*, low-contrast boundaries) from the decoder’s last three multi-scale layers (High: H×W, Mid: H/2×W/2, Low: H/4×W/4) while avoiding cross-category interference. It first splits each scale’s decoder feature map $F_{\text{dec}}^{k}$ (where *k* = 1,2,3 denotes scale, $D_{k}$ is total channels) into *C* category-specific groups:


$$\begin{array}{c} F_{\text{split},c}^{k}=F_{\text{dec}}^{k}\left[:,\;s_{c,k}\;:\;e_{c,k},:,:,\right], \end{array}$$ (2)

where $s_{c,k}$ and $e_{c,k}$ denote the start and end channel indices of the *c* -th category-specific feature group at the *k* -th scale, respectively. For index assignment: when 1≤c≤C−1, $s_{c,k}=R\cdot (c-1)$ and $e_{c,k}=R\cdot c$ (with $R=\lfloor D_{k}/C\rfloor$ representing the base number of channels per group); when *c* = *C*, $s_{c,k}=R\cdot (C-1)$ and $e_{c,k}=D_{k}$ (covering all remaining channels). This design ensures no channels are discarded due to the potential indivisibility of $D_{k}$ (total channels of $F_{\text{dec}}^{k}$) by *C* (number of tissue categories), while achieving effective feature isolation between different categories.

Next, adaptive weights for each category group are generated via a simplified smooth function applied to the Seg-Head’s probability map $Y^{\prime }$ (interpolated to match $F_{\text{dec}}^{k}$‘s resolution), which uses a sharp central peak (for medium-confidence ambiguous regions) and flat baseline (for reliable regions) to balance enhancement and preservation, expressed as:


$$\begin{array}{c} W_{c}^{k}=\exp\left(-30\cdot ((Y^{\prime }{)}_{c}^{k}-0.5{)}^{4}\right)+0.3\cdot \sigma \left(20\cdot \left(0.5-|(Y^{\prime }{)}_{c}^{k}-0.5|\right)\right). \end{array}$$ (3)

Here, σ(·) denotes the Sigmoid activation function, whose mathematical definition is given by $\sigma (x)=\frac{1}{1+\exp(-x)}$, *u* represents the input probability value (*e.g.*, the category probability ${Y^{\prime k}}_{:,:,c}$ output by the Segmentation Head) and ranges within the interval [0,1].

Finally, each category group $F_{\text{split},c}^{k}$ is element-wise multiplied by $W_{c}^{k}$ to get enhanced groups $F_{\text{enh},c}^{k}$, which are concatenated to form the final enhanced feature map $F_{e}^{k}$ for each scale.

The category-channel mapping in DICE explicitly aligns feature groups with specific tissue types (ICM, TE, blastocoel, ZP), ensuring that structural enhancement respects biological layering. This design reflects the inherent nesting of blastocyst tissues, allowing the network to strengthen features that define inter-tissue boundaries while preserving intra-tissue continuity.

#### Multi-scale feature aggregation (MSFA) module.

The Multi-Scale Feature Aggregation (MSFA) module aggregates multi-scale enhanced features (High-scale *F*_1_, Mid-scale *F*_2_, Low-scale *F*_3_) to capture hierarchical structural cues for blastocyst tissue segmentation. First, it performs resolution alignment by bilinearly upsampling Mid-scale *F*_2_ and Low-scale *F*_3_ to match the High-scale resolution (H×W), yielding upsampled features *F*_2,*up*_ and *F*_3,*up*_. Next, it conducts attention-weighted fusion, where channel attention weights ($\alpha_{1},\alpha_{2},\alpha_{3}$) calculated via a Sigmoid map are assigned to *F*_1_, *F*_2,*up*_, and *F*_3,*up*_ to adaptively adjust their feature importance, resulting in weighted features as expressed in:


$$\begin{array}{c} F_{w1}=\alpha_{1}⊙F_{1},\;F_{w2}=\alpha_{2}⊙F_{2,up},\;F_{w3}=\alpha_{3}⊙F_{3,up}. \end{array}$$ (4)

Finally, it executes concatenation and regression. The weighted features $F_{w1}$, $F_{w2}$, and $F_{w3}$ are concatenated into a single aggregated feature map $F_{concat}$, which is then fed into a regression layer composed of 3×3 convolution blocks and a 1×1 convolution. This regression layer applies Sigmoid activation to map the output to the interval [0,1], generating the structural probability map $S^{\prime }$ as formulated in


$$\begin{array}{c} S^{\prime }=\text{Regression}(F_{concat}). \end{array}$$ (5)

#### Intuitive role of topology guidance in semi-supervised learning.

Topology guidance provides an additional source of anatomical regularization that complements conventional consistency-based SSL. For unlabeled images, where no ground truth masks are available, the model must rely on internal consistency between differently augmented views. However, pixel-wise consistency alone can perpetuate structural errors (*e.g.*, fragmented tissues or physically impossible layer relationships). Our topology-guided head introduces a geometric prior: it enforces that predictions should conform to the nested organization of blastocyst tissues. By requiring the two backbones to produce structurally coherent probability maps, where signed distances to boundaries evolve smoothly and layers remain properly nested. The model learns to extrapolate anatomical from limited labeled examples to the unlabeled data. This mechanism acts as a form of “structural common sense”, reducing the reliance on large labeled datasets while improving segmentation plausibility.

### Loss function

The total loss of the network combines a supervised loss for labeled data and an unsupervised consistency loss.

#### Supervised Loss (${ℒ}_{sup}$).

For labeled data (*X*, *Y*, *S*), ${ℒ}_{sup}$ includes three components: Pixel cross entropy (CE) loss (${ℒ}_{CE}$): Aligns $Y^{\prime }$ with *Y*:


$$\begin{array}{c} {ℒ}_{CE}=-\frac{1}{H\times W}\sum_{i=1}^{H\times W}\sum_{c=1}^{C}Y_{i,c}\cdot \log(Y_{i,c}^{\prime }), \end{array}$$ (6)

where $Y_{i,c}$ is the one-hot label of the *i*-th pixel for category *c*.

Structural mean-square error (MSE) loss (${ℒ}_{struct-MSE}$): Aligns $S^{\prime }$ with structural ground truth *S*:


$$\begin{array}{c} {ℒ}_{struct-MSE}=\frac{1}{H\times W}\sum_{i=1}^{H\times W}(S_{i}^{\prime }-S_{i}{)}^{2}, \end{array}$$ (7)

where $S_{i}^{\prime }$ and $S_{i}$ are values of $S^{\prime }$ and *B* at the *i*-th pixel.

Structure consistency loss (${ℒ}_{struct-consis}$): ensures consistency between $S^{\prime }$ of the two backbones ($S_{1}^{\prime },S_{2}^{\prime }$):


$$\begin{array}{c} {ℒ}_{struct-consis}=\frac{1}{H\times W}\sum_{i=1}^{H\times W}(S_{1,i}^{\prime }-S_{2,i}^{\prime }{)}^{2}. \end{array}$$ (8)

The weighted supervised loss is:


$$\begin{array}{c} {ℒ}_{sup}={ℒ}_{CE}+\alpha \cdot {ℒ}_{struct-MSE}+\beta \cdot {ℒ}_{struct-consis}, \end{array}$$ (9)

where α=0.002, β=0.001 (determined via cross-validation).

#### Unsupervised Consistency Loss (${ℒ}_{unsup}$).

For unlabeled data $X_{u}$, ${ℒ}_{unsup}$ enforces consistency between the two backbones:


$$\begin{array}{c} {ℒ}_{unsup}=\frac{1}{H\times W}\sum_{i=1}^{H\times W}(S_{1,i}^{\prime }-S_{2,i}^{\prime }{)}^{2}. \end{array}$$ (10)

#### Total Loss with Dynamic Self-Distillation Weight.

The final total loss ${ℒ}_{total}$ integrates the supervised loss (from labeled data) and unsupervised self-distillation loss (from unlabeled data), leveraging the independent weight design of *B*_0_ and *B*_1_ to achieve synergistic learning:


$$\begin{array}{c} {ℒ}_{\text{total}}={ℒ}_{\text{sup}}+\gamma *{ℒ}_{\text{unsup}}, \end{array}$$ (11)

where γ follows the setting of the previous work [17].

## Experiments and results

### Datasets

To validate the effectiveness and generalization of the proposed method, two publicly available datasets with different medical imaging modalities and structural characteristics are used for evaluation.

The first dataset is a human blastocyst microscopic image dataset [18], which serves as the primary testbed for blastocyst segmentation tasks. These images were acquired using Hoffman modulation contrast optics, a specific type of phase-contrast microscopy commonly used in time-lapse embryo culture systems to visualize transparent specimens without staining. It comprises 249 high-resolution blastocyst images paired with manually annotated ground truth (GT) masks, where annotations for key blastocyst components-inner cell mass and blastocoel-are provided by the Pacific Center for Reproductive Medicine. For this dataset, 190 images of the data are used for training, 9 images for validation, and 50 images for testing. For semi-supervised learning, 10% and 50% of the data in the training set are used for training, respectively.

The second dataset is the left atrium 3D magnetic resonance imaging (MRI) dataset [19]. Specifically, these are gadolinium-enhanced MRI (GE-MRI) scans, which provide high soft-tissue contrast for atrial structures. As a widely used benchmark for semi-supervised segmentation performance evaluation [17,20–26], it contains 100 labeled 3D GE-MRI scans of the left atrium. To ensure comparability with the existing literature, we follow the data splitting protocol established in [24,26].

### Implementation details

To alleviate overfitting and improve the generalization of the model to diverse input variations, a suite of data augmentation strategies is applied during training, including random scaling (to simulate different imaging magnifications), random rotation (to account for arbitrary image orientations), and adaptive adjustments to brightness and contrast (to mimic real-world variations in imaging conditions). For training hyperparameters, a carefully tuned configuration is adopted: the training process runs for a total of 2000 epochs to ensure sufficient convergence; the initial learning rate is set to $1\times 10^{-4}$ to balance fast convergence and stable parameter updates; the weight decay of $5\times 10^{-5}$ is introduced to regularize the model and prevent excessive parameter growth. For blastocyst segmentation, four standard metrics from prior literature [18] are adopted: Accuracy, Recall, Dice Coefficient, and Jaccard Index. The U-Net architecture [27] is selected as the backbone. For LA segmentation, the evaluation focuses on metrics suitable for 3D structural analysis: Dice Coefficient, Jaccard Index (for overlap assessment), average surface distance (ASD), and 95% Hausdorff Distance (95HD) [26] (for boundary proximity measurement). Here, the VNet [28] serves as the backbone.

### Comparison with state-of-the-art methods

From Table 1, compared to other semi-supervised methods, it is clear that in the ICM binary segmentation task with 10% labeled data, TGDBN achieves an Accuracy of 97.62%, a Dice coefficient of 91.78%, and a Jaccard index of 86.33%, all of which are the optimal results in this data partition. These metrics reflect a high level of spatial overlap between TGDBN’s segmentation results and ground truth, indicating its ability to precisely delineate the ICM. When the labeled data ratio increases to 50%, TGDBN still performs exceptionally well.

**Table 1:** Comparison of performances of semi-supervised learning methods with U-Net as the backbone for binary segmentation. “*” and “**” denote significance levels of p≤0.05 and p≤0.01, respectively, from a two-sided paired t-test when comparing our best model with others. Best results are highlighted in bold.

Method	ICM (10% Labeled)				ICM (50% Labeled)			
	Accuracy (%) ↑	Recall (%) ↑	Dice (%) ↑	Jaccard (%) ↑	Accuracy (%) ↑	Recall (%) ↑	Dice (%) ↑	Jaccard (%) ↑
SupOnly	96.96$\pm 0.02^{**}$	87.08$\pm 1.14^{*}$	87.59$\pm 0.47^{**}$	81.32$\pm 0.48^{**}$	97.99$\pm 0.03^{**}$	91.44$\pm 0.48^{**}$	91.42$\pm 0.31^{**}$	86.95$\pm 0.23^{**}$
MT [17]	97.51±0.11	90.16$\pm 0.52^{*}$	90.20$\pm 0.41^{**}$	84.80$\pm 0.61^{*}$	98.11$\pm 0.07^{*}$	92.33$\pm 0.33^{*}$	92.42$\pm 0.34^{**}$	88.01$\pm 0.37^{**}$
DAN [20]	96.89$\pm 0.14^{*}$	91.22$\pm 0.18^{*}$	89.54$\pm 0.17^{**}$	83.13$\pm 0.18^{**}$	97.99$\pm 0.06^{**}$	**94.23** ±0.68	93.24$\pm 0.19^{*}$	88.46$\pm 0.12^{**}$
UAMT [21]	96.86$\pm 0.31^{*}$	90.79$\pm 0.01^{*}$	89.01$\pm 0.84^{*}$	82.85$\pm 1.10^{*}$	98.11$\pm 0.05^{**}$	93.19±0.63	92.97$\pm 0.44^{**}$	88.49$\pm 0.44^{**}$
AEM [22]	97.47$\pm 0.10^{*}$	91.41±1.41	90.76$\pm 0.58^{*}$	85.23$\pm 0.75^{*}$	98.06$\pm 0.10^{*}$	94.18±0.64	93.34±0.14	88.83$\pm 0.17^{*}$
RD [23]	97.31$\pm 0.10^{*}$	90.33±1.17	90.23$\pm 0.83^{*}$	84.33$\pm 0.92^{*}$	98.04$\pm 0.15^{*}$	92.11±1.20	91.81$\pm 0.72^{*}$	87.51$\pm 1.00^{*}$
DTC [24]	97.07$\pm 0.05^{**}$	91.40±1.45	90.37$\pm 0.52^{**}$	83.99$\pm 0.60^{**}$	97.92$\pm 0.09^{*}$	92.87±0.71	92.09$\pm 0.83^{*}$	87.49$\pm 0.79^{*}$
ICT [25]	97.50±0.25	91.28±0.35	91.12±0.47	85.43±0.73	98.10$\pm 0.02^{*}$	93.02$\pm 0.23^{**}$	93.15$\pm 0.24^{*}$	88.61$\pm 0.20^{**}$
MCF [29]	97.48±0.12	90.83$\pm 0.98^{*}$	90.93$\pm 0.51^{*}$	85.15$\pm 0.47^{*}$	98.16±0.12	93.40$\pm 0.37^{*}$	93.26$\pm 0.40^{*}$	88.83$\pm 0.60^{*}$
ACMT [26]	95.88$\pm 0.26^{**}$	89.13$\pm 1.12^{*}$	86.84$\pm 0.30^{**}$	79.57$\pm 0.41^{**}$	97.89$\pm 0.23^{*}$	93.23±1.19	92.41±1.33	87.87$\pm 1.23^{*}$
Unimatch [30]	97.34$\pm 0.08^{**}$	**92.78** ±0.33*	91.17$\pm 0.17^{**}$	85.32$\pm 0.21^{**}$	98.00$\pm 0.01^{**}$	94.11±0.22	92.99$\pm 0.17^{*}$	88.16$\pm 0.18^{**}$
LeFeD [31]	97.47±0.05	90.78±0.40	90.86$\pm 0.29^{**}$	85.18$\pm 0.34^{**}$	98.23±0.09	93.23$\pm 0.53^{*}$	93.75$\pm 0.29^{*}$	89.40$\pm 0.47^{*}$
TGDBN (Ours)	**97.62** ±0.07	92.49±0.38	**91.78** ±0.29	**86.33±0.22**	**98.28** ±0.05	94.03±0.27	**94.01±0.32**	**89.78±0.37**
SupOnly (Upper Bound)	98.46±0.06	95.52±0.40	95.00±0.09	91.06±0.16	98.46±0.06	95.52±0.40	95.00±0.09	91.06±0.16

**Fig 4:**
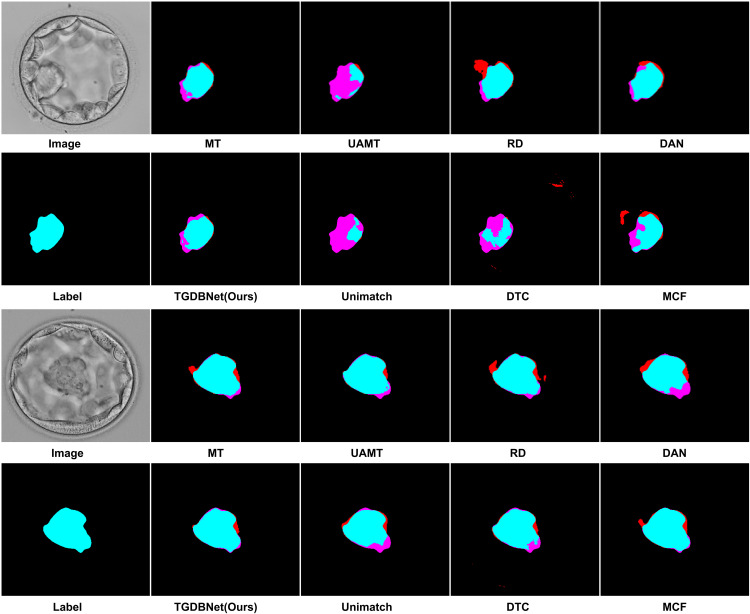
Visualization results of our method and other state-of-the-art methods in ICM segmentation task.

The qualitative visualization results in Fig 4 further confirm the superiority of TGDBN. Its predicted segmentation masks closely match the ground truth labels, while other semi-supervised methods show scattered and less coherent results. Notably, TGDBN excels in handling the boundaries of blastocyst components, where errors from competing methods are mainly concentrated in low-contrast and small-scale regions. These qualitative findings collectively validate the effectiveness of our proposed method, especially in its robust boundary delineation capability.

### Ablation study

Table 2 displays the ablation study outcomes for the ICM dataset, where our proposed TGDBN maintains consistent superiority over all baseline models. The “B” baseline delivers acceptable performance across all evaluation metrics but remains relatively underwhelming compared to other variants. Even when augmented with individual functional modules, such as “DICE” in the “B + D” variant, or “MSFA” in the “B + M” variant, the performance sees incremental improvements, yet still fails to match TGDBN. This confirms that the integrated design of TGDBN, which combines pixel-level details with topological structural guidance, is the key to achieving more precise and robust ICM segmentation results.

**Table 2:** Ablation study results on ICM dataset. “*” and “**” denote significance levels of p≤0.05 and p≤0.01, respectively, from a two-sided paired t-test when comparing our best model with others. Best results are highlighted in bold.

B	D	M	Accuracy (%) ↑	Recall (%) ↑	Dice (%) ↑	Jaccard (%) ↑
✓			96.96$\pm 0.02^{**}$	87.08$\pm 1.14^{*}$	87.59$\pm 0.47^{**}$	81.32$\pm 0.48^{**}$
✓	✓		97.48±0.03	91.58±0.67	91.15$\pm 0.25^{*}$	85.34$\pm 0.31^{**}$
✓		✓	97.54±0.04	92.00±0.97	91.21±0.62	85.63$\pm 0.42^{*}$
✓	✓	✓	**97.62±0.07**	**92.49** ±0.38	**91.78±0.29**	**86.33±0.22**

**Fig 5:**
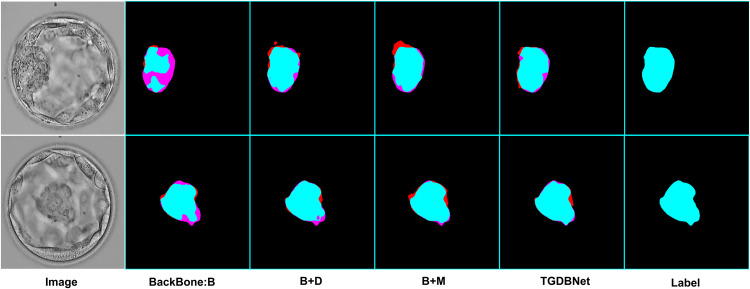
Visualization results of ablation study in ICM segmentation task.

The qualitative visualization results in Fig 5 further highlight the value of topological structure guidance in TGDBN. Our method’s segmentation outputs closely align with the ground truth, effectively preserving and accurately depicting the inherent topological architecture of the ICM. By contrast, variants lacking this integrated guidance struggle to capture such structural nuances, underscoring TGDBN’s unique advantage in leveraging topological cues for high-fidelity segmentation.

## Discussions

Our experiments demonstrate that explicit topological guidance substantially improves semi-supervised segmentation, particularly under low-label regimes. The signed distance representation used in our structural probability map directly encodes the layered organization of blastocyst tissues, providing a continuous geometric signal that is both differentiable and biologically interpretable. Unlike topological invariants (*e.g.*, Betti numbers) or graph-based constraints, our approach integrates topological reasoning into the feature learning process, allowing dynamic interaction between pixel-level segmentation and structural optimization. This design not only mitigates boundary ambiguity but also enables the model to leverage unlabeled data more effectively by enforcing anatomical consistency across predictions.

**Fig 6:**
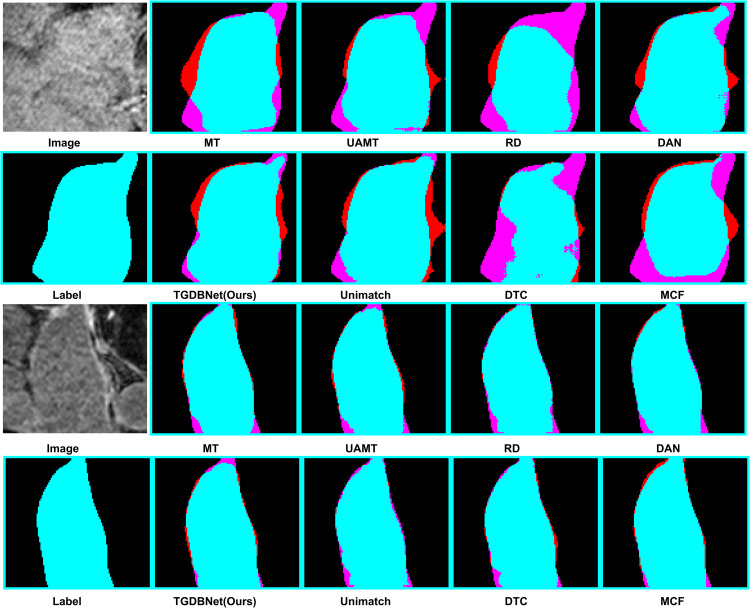
Visualization results on the LA dataset.

To further verify the generalization of our method, we conducted validation on the LA dataset-a benchmark with anatomical structural similarities to blastocysts. Table 3 compares semi-supervised segmentation methods for LA binary tasks, all using V-Net as the backbone, and our TGDBN stands out with exceptional performance. Whether under 10% or 20% labeled data ratios, TGDBN maintains strong competitiveness. This consistent performance across both low and high labeled data scenarios confirms its capability to mine valuable information from unlabeled data, thereby ensuring accurate LA segmentation. Fig 6 provides qualitative comparisons of LA segmentation results between TGDBN and other semi-supervised methods, offering direct visual evidence of TGDBN’s superiority. This visual validation not only aligns with the quantitative metrics in Table 3 but also demonstrates TGDBN’s stronger ability to capture the unique features of LA images. It further corroborates the performance insights discussed earlier, serving as additional proof of our proposed method’s effectiveness.

**Table 3:** Comparison of performances of semi-supervised learning methods with V-Net as the backbone for binary segmentation on LA dataset. “*” and “**” denote significance levels of p≤0.05 and p≤0.01, respectively, from a two-sided paired t-test when comparing our best model with others. Best results are highlighted in bold.

Method	LA (10% Labeled)				LA (20% Labeled)			
	Dice (%) ↑	Jaccard (%) ↑	ASD (voxel) ↓	95HD (voxel) ↓	Dice (%) ↑	Jaccard (%) ↑	ASD (voxel) ↓	95HD (voxel) ↓
SupOnly	75.72$\pm 1.06^{**}$	65.64$\pm 1.37^{**}$	29.54$\pm 1.94^{**}$	7.55$\pm 1.67^{*}$	86.13$\pm 0.27^{**}$	76.79$\pm 0.11^{**}$	20.57$\pm 3.69^{*}$	5.02$\pm 1.57^{*}$
MT [17]	87.71$\pm 0.62^{*}$	78.89$\pm 0.69^{*}$	12.50$\pm 0.97^{*}$	3.48$\pm 0.18^{**}$	90.08$\pm 0.24^{**}$	82.27$\pm 0.32^{**}$	9.66±3.09	2.72±0.62
DAN [20]	88.83$\pm 0.21^{*}$	80.31±0.40	10.19±1.70	2.74±0.19	91.23$\pm 0.23^{*}$	84.08$\pm 0.39^{*}$	6.77$\pm 0.49^{*}$	1.96$\pm 0.06^{**}$
UAMT [21]	88.21±0.70	79.50±0.90	10.01±1.62	3.72±0.83	90.99$\pm 0.13^{**}$	83.66$\pm 0.20^{**}$	7.20$\pm 0.71^{*}$	2.38$\pm 0.38^{*}$
AEM [22]	87.67$\pm 0.60^{*}$	78.35$\pm 0.95^{*}$	16.95±6.63	4.40$\pm 1.90^{*}$	90.18$\pm 0.53^{*}$	82.45$\pm 0.78^{*}$	11.53±5.30	2.77±0.94
RD [23]	86.72$\pm 0.48^{*}$	77.10$\pm 0.61^{**}$	23.45±8.45	6.37±1.95	89.83$\pm 0.40^{**}$	81.83$\pm 0.58^{**}$	9.63±3.13	3.29$\pm 0.88^{*}$
DTC [24]	87.99$\pm 0.22^{**}$	78.97$\pm 0.32^{**}$	11.24±2.55	2.83±0.24	90.90$\pm 0.22^{**}$	83.58$\pm 0.40^{**}$	6.67$\pm 0.24^{**}$	2.13$\pm 0.01^{**}$
ICT [25]	88.22$\pm 0.17^{*}$	79.22$\pm 0.25^{*}$	12.83$\pm 1.12^{*}$	2.68±0.19	91.22$\pm 0.10^{**}$	84.04$\pm 0.17^{**}$	7.11$\pm 0.18^{**}$	1.99$\pm 0.07^{**}$
MCF [29]	86.58$\pm 0.62^{*}$	76.96$\pm 0.83^{*}$	17.65±6.02	3.77±1.21	90.62$\pm 0.14^{**}$	83.18$\pm 0.28^{**}$	8.59$\pm 1.07^{*}$	2.04$\pm 0.21^{*}$
ACMT [26]	87.89$\pm 0.16^{**}$	79.09$\pm 0.07^{**}$	10.22±1.49	3.29$\pm 0.32^{*}$	91.21$\pm 0.08^{**}$	84.02$\pm 0.09^{**}$	6.38$\pm 0.29^{*}$	2.19$\pm 0.09^{**}$
Unimatch [30]	88.01$\pm 0.58^{*}$	78.82$\pm 0.87^{*}$	12.08$\pm 0.89^{*}$	3.03$\pm 0.18^{*}$	91.31$\pm 0.25^{*}$	84.08$\pm 0.42^{*}$	9.35$\pm 2.11^{*}$	2.11$\pm 0.13^{**}$
LeFeD [31]	87.93$\pm 0.24^{*}$	78.83$\pm 0.35^{**}$	11.45$\pm 1.62^{*}$	2.45±0.20	91.94±0.31	85.29±0.50	5.76$\pm 1.65^{**}$	1.65±0.09
TGDBN (Ours)	**89.47±0.16**	**81.27±0.28**	**8.09±0.46**	**2.45±0.04**	**92.32±0.04**	**85.83±0.07**	**5.30±0.19**	**1.61±0.07**
SupOnly (Upper Bound)	93.60±0.05	88.01±0.09	4.40±0.07	1.41±0.03	93.60±0.05	88.01±0.09	4.40±0.07	1.41±0.03

## Conclusions

This study tackles two fundamental challenges in semi-supervised medical image segmentation: achieving sufficient accuracy under low-labeling conditions and effectively leveraging topological structure information. Taking TGDBN as the solution, its effectiveness was validated through two typical medical segmentation tasks. In the ICM segmentation task, based on a dataset containing 249 microscopic images, TGDBN achieved significantly improved performance compared to baselines under 10%–50% labeled data ratios. Qualitative results also showed that it could accurately capture the topological structure of the ICM and avoid the scattered missegmentation issues common in other methods. In the LA segmentation task, using a dataset of 100 3D MRI scans and V-Net as the backbone network, TGDBN still exhibited excellent performance with 10%–20% labeled data, effectively mitigating problems such as boundary blurriness and regional missegmentation to a certain extent. The advantages of TGDBN stem from its integrated design of “pixel-level constraints + topological guidance” ，which enables efficient extraction of information from unlabeled data. It is compatible with 2D/3D multimodal medical data and demonstrates strong generalization. However, further verification and research are required to explore its performance under more limited labeling conditions and reduce its computational resource overhead.
